# A human milk oligosaccharide prevents intestinal inflammation in adulthood via modulating gut microbial metabolism

**DOI:** 10.1128/mbio.00298-24

**Published:** 2024-03-05

**Authors:** Kasey M. Schalich, Matthew A. Buendia, Harpreet Kaur, Yash A. Choksi, M. Kay Washington, Gabriela S. Codreanu, Stacy D. Sherrod, John A. McLean, Richard M. Peek, Jr., Sari A. Acra, Steven D. Townsend, Fang Yan

**Affiliations:** 1 Department of Pediatrics, Vanderbilt University Medical Center, Nashville, Tennessee, USA; 2 Department of Medicine, Vanderbilt University Medical Center, Nashville, Tennessee, USA; 3 Department of Pathology, Microbiology and Immunology, Vanderbilt University Medical Center, Nashville, Tennessee, USA; 4 Department of Chemistry, Vanderbilt University, Nashville, Tennessee, USA; 5 Center for Innovative Technology, Vanderbilt University, Nashville, Tennessee, USA; 6 Department of Cell and Developmental Biology, Vanderbilt University, Nashville, Tennessee, USA; Institut Pasteur, Paris, France

**Keywords:** 2′-fucosyllactose, *Bifidobacterium longum *subsp*. infantis*, human milk oligosaccharide, inflammatory bowel diseases, intestinal barrier, pantothenate, the gut microbiota

## Abstract

Observational evidence suggests that human milk oligosaccharides (HMOs) promote the growth of commensal bacteria in early life and adulthood. However, the mechanisms by which HMOs benefit health through modulation of gut microbial homeostasis remain largely unknown. 2′-fucosyllactose (2′-FL) is the most abundant oligosaccharide in human milk and contributes to the essential health benefits associated with human milk consumption. Here, we investigated how 2′-FL prevents colitis in adulthood through its effects on the gut microbial community. We found that the gut microbiota from adult mice that consumed 2′-FL exhibited an increase in abundance of several health-associated genera, including *Bifidobacterium* and *Lactobacillus*. The 2′-FL-modulated gut microbial community exerted preventive effects on colitis in adult mice. By using *Bifidobacterium infantis* as a 2′-FL-consuming bacterial model, exploratory metabolomics revealed novel 2′-FL-enriched secretory metabolites by *Bifidobacterium infantis*, including pantothenol. Importantly, pantothenate significantly protected the intestinal barrier against oxidative stress and mitigated colitis in adult mice. Furthermore, microbial metabolic pathway analysis identified 26 dysregulated metabolic pathways in fecal microbiota from patients with ulcerative colitis, which were significantly regulated by 2′-FL treatment in adult mice, indicating that 2′-FL has the potential to rectify dysregulated microbial metabolism in colitis. These findings support the contribution of the 2′-FL-shaped gut microbial community and bacterial metabolite production to the protection of intestinal integrity and prevention of intestinal inflammation in adulthood.

**IMPORTANCE:**

At present, neither basic research nor clinical studies have revealed the exact biological functions or mechanisms of action of individual oligosaccharides during development or in adulthood. Thus, it remains largely unknown whether human milk oligosaccharides could serve as effective therapeutics for gastrointestinal-related diseases. Results from the present study uncover 2′-FL-driven alterations in bacterial metabolism and identify novel *B. infantis*-secreted metabolites following the consumption of 2′-FL, including pantothenol. This work further demonstrates a previously unrecognized role of pantothenate in significantly protecting the intestinal barrier against oxidative stress and mitigating colitis in adult mice. Remarkably, 2′-FL-enhanced bacterial metabolic pathways are found to be dysregulated in the fecal microbiota of ulcerative colitis patients. These novel metabolic pathways underlying the bioactivities of 2′-FL may lay a foundation for applying individual oligosaccharides for prophylactic intervention for diseases associated with impaired intestinal homeostasis.

## INTRODUCTION

Human milk oligosaccharides (HMOs) contain more than 200 structurally diverse bioactive components and constitute the third most abundant solid component in human milk (1). The human cells lining the gastrointestinal tract do not possess the enzymatic machinery that is required for metabolizing HMOs, and thus HMOs can reach the colon intact (2). HMOs can be metabolized by gut commensal bacteria that possess a gene cluster that encodes various oligosaccharide transporters and glycosyl hydrolases (3), such as *Bifidobacteria*, *Bacteroides*, *Lactobacillus*, and a variety of other firmicutes (4). One well-recognized benefit of HMOs is to function as prebiotics to promote the growth of commensal bacteria in infancy (5). The beneficial effects of HMOs on the gut microbial community across the lifespan have also been revealed. HMOs can promote engraftment of *Bifidobacterium longum* subspecies *infantis* (*B. infantis*) in the gut microbiota community in healthy adults without antibiotic treatment (6). Moreover, a clinical study found that an individual HMO, 2′-fucosyllactose (2′-FL), is safe and well tolerated and increases the relative abundance of *Bifidobacterium* in healthy adults (7). Animal studies reported that 2′-FL could augment *Bifidobacterium pseudocatenulatum* persistence and its ability to alleviate colitis in adult mice (8). Several other beneficial effects of HMOs have been reported. HMOs are associated with fostering anti-infective activity (9) and promoting immune system development (10) in early life, while supplementation with 2′-FL has been found to reduce intestinal inflammation, promote mucus production, and increase epithelial barrier integrity in adult rodent models of colitis (11, 12). Elucidating the mechanisms underlying the bioactivities of individual oligosaccharides in human milk is essential to clearly define the health benefits of HMOs. 2′-FL is the most abundant oligosaccharide in milk from mothers with the Se locus (1) but is absent in cow’s milk (13). Although the correlation between 2′-FL and its beneficial health effects has been recognized, the mechanism(s) by which 2′-FL protects intestinal homeostasis remain unclear. Particularly, the contribution of the 2′-FL and HMO-modulated gut microbial community to the biological effects in infancy and adulthood remains largely unclear.

The mutualistic relationship between the gut microbiota and the host establishes a nutrient-rich and metabolically favorable environment for the microbiota and confers health benefits to the host, such as facilitating the development of the gastrointestinal tract, induction of immunotolerance in early life (14, 15), and maintaining functions of the immune system in adulthood (16). Dysbiosis of the gut microbiota has been identified as contributing to and/or a consequence of pathological factors in several diseases, including inflammatory bowel diseases (IBD) (17). IBD, including Crohn’s disease (CD) and ulcerative colitis (UC), are a group of idiopathic recurrent inflammatory illnesses of the gastrointestinal tract. IBD has become a public health challenge, with accelerating incidence worldwide (18). New therapeutic strategies for IBD are needed because the commonly used treatment for IBD, anti-tumor necrosis factor (TNF) agents, generally induce sustained remission in less than half of patients (19). Manipulation of the gut microbiota is currently being investigated as a potential strategy to prevent and treat IBD (20). Therefore, this study focused on investigating how 2′-FL prevents colitis through modulating gut microbial metabolism in adulthood and identifying if 2′-FL has the potential to rectify the dysregulated microbial metabolism in IBD patients.

## RESULTS

### 2′-FL-modulated gut microbial profile contributes to the prevention of colitis in adult mice

**Fig 1:**
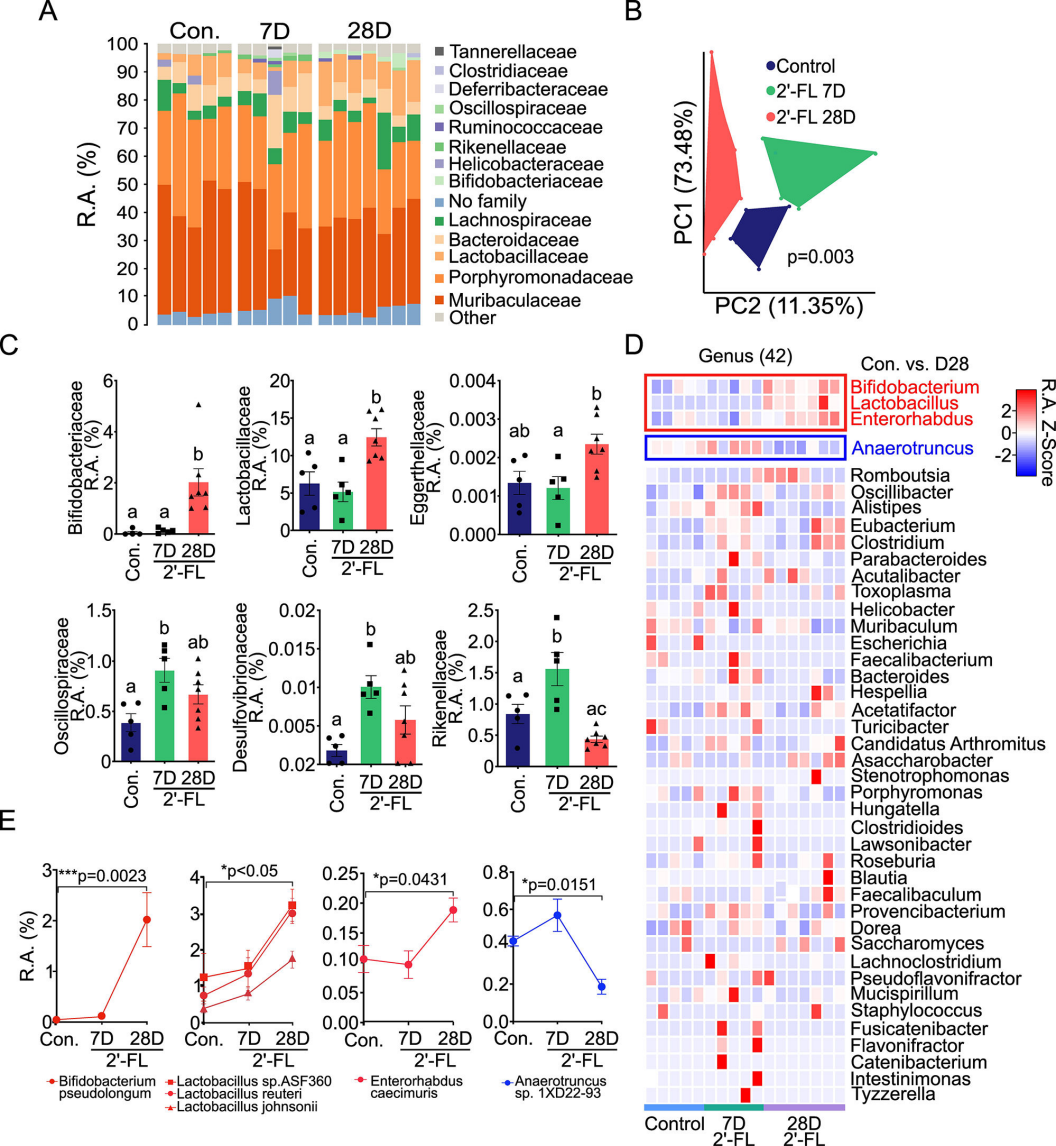
2′-FL modifies the gut microbial composition in adult mice. Adult mice received 2′-FL (1 mg/mL) in drinking water for 7 (7D, *n* = 5) or 28 (28D, *n* = 7) days. Mice receiving regular drinking water only were used as the control (Con., *n* = 5). The fecal bacteria were analyzed by WGS sequencing. (A) Relative abundance (R.A.%) taxa plot depicting the relative abundance of the top 15 families of 2′-FL consumption. (B) The weighted unifrac PCoA coordinates for the three groups. (C) 2′-FL-induced alterations in the R.A.% of the *Bifidobacteriaceae*, *Lactobacillaceae*, *Eggerthellaceae*, *Oscillospiraceae*, *Desulfovibrionaceae*, and *Rikenellaceae* families compared to the control group. (D) Heatmap illustrating the relative abundance *Z* score for all 42 identified genera across the control, 7D 2′-FL, and 28D 2′-FL groups. (E) Species that were significantly (*P* < 0.05) upregulated (*Lactobacillus*, *Bifidobacterium*, and *Enterorhabdus*) or downregulated (*Anaerotruncus*) in the 28D 2′-FL group compared to the control group within the four significantly altered genera were identified. For panel C, different letters between groups (a, b, or c) indicate that the two groups are significantly different; any common letters between groups indicate that they are not significantly different.

Bacteria that can metabolize HMOs, such as *B. infantis* and *Bifidobacterium breve,* are abundant in breastfed infants (21), which persist into adulthood in decreasing but stable abundance (22, 23). To determine the exact impact of 2′-FL on the gut microbial composition in adult mice, whole-genome sequencing of fecal bacteria from mice treated with 2′-FL was performed. A relative abundance taxa plot depicts the top 15 families in control (Con.), 2′-FL 7 days (7D), and 2′-FL 28 days (28D) treatment groups (Fig. 1A). 2′-FL induced the unweighted unifrac principal coordinates analysis (PCoA) coordinates to significantly shift between the three groups (*P* = 0.003) (Fig. 1B). The relative abundance of several families was altered in the 2′-FL-treated groups compared to the control group (Fig. 1C) (Table S1). The relative abundances of *Bifidobacteriaceae* and *Lactobacillaceae* were quantitatively most enriched in the 2′-FL 28D group compared to the control (*P* = 0.0074 and *P* = 0.0126, respectively) and the 2′-FL 7D groups (*P* = 0.0095 and *P* = 0.0039, respectively). The relative abundance of *Eggerthellaceae* in 2′-FL-28D group was increased without significance compared to the control (*P* = 0.0567). The *Oscillospiraceae*, *Rikenellaceae*, and *Desulfovibrionaceae* families demonstrated a similar pattern by increasing significantly in the 2′-FL 7D group compared to the control group (*P* = 0.0115, *P* = 0.0244, and *P* = 0.0097, respectively), but decreased in the 2′-FL 28D group with no significant difference as compared to the control group.

Of the 42 genera identified, three were significantly enriched in the 2′-FL 28D group, but not in the 2′-FL-7D group, compared to the control group: *Bifidobacterium* (*P* = 0.0028), *Lactobacillus* (*P* = 0.0048), and *Enterorhabdus* (*P* = 0.0220). In contrast, the genus *Anaerotruncus* displayed a significant decrease in relative abundance (*P* = 0.0059) (Fig. 1D; Table S2). Next, individual species that were contributing to the specific genus- and family-level enrichment with 2′-FL consumption were identified (Table S3). *Bifidobacterium pseudolongum* (*P* = 0.0023), *Lactobacillus* sp. ASF360*, reurteri,* and *johnsonii* (*P* < 0.05 for each), and *Enterorhabdus caecimuris P* = 0.0431) were significantly enriched, and *Anaerotruncus* sp*.* 1XD22-93, *P* = 0.0151 was significantly diminished in 2′-FL 28D compared to the control groups. No significant changes in these four species were identified in the 2′-FL 7D group (Fig. 1E).

**Fig 2:**
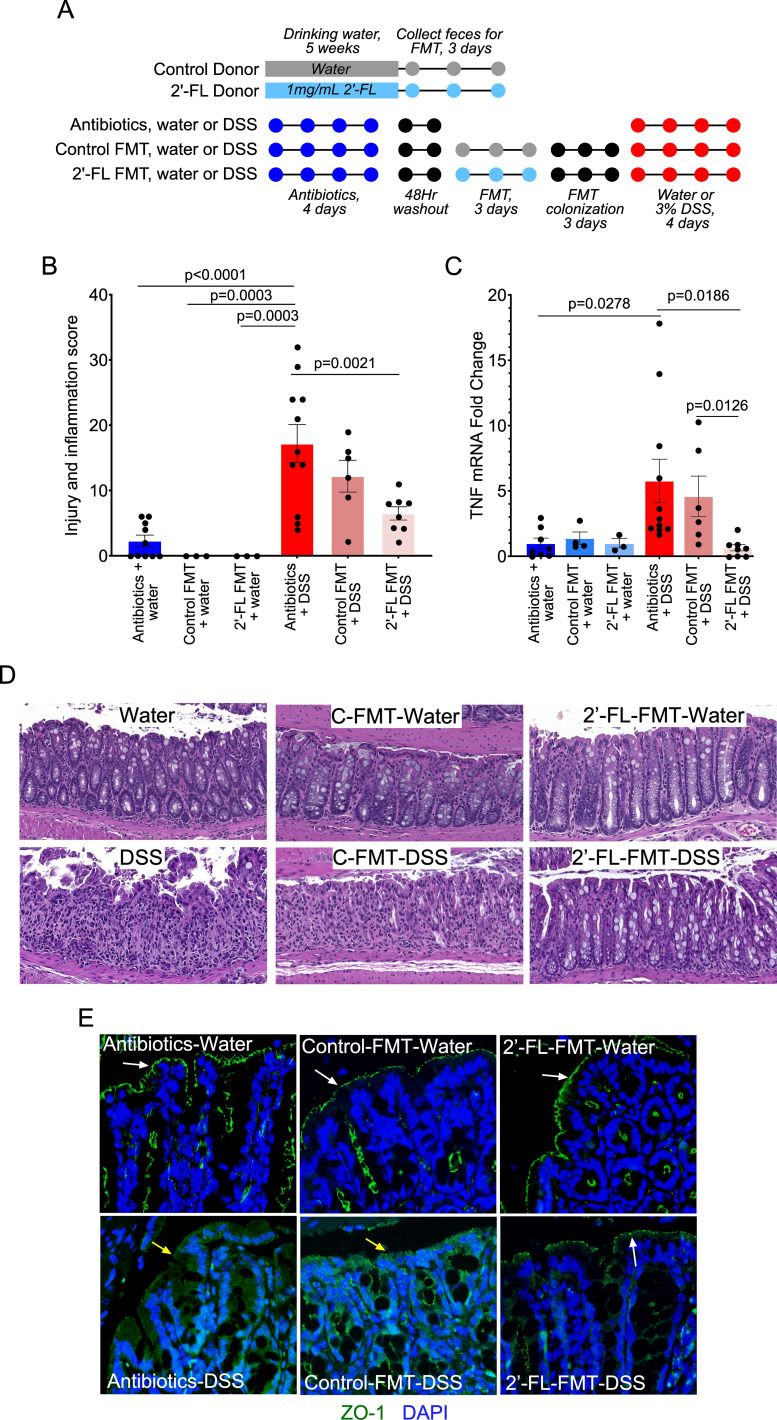
2′-FL-modulated gut microbial community plays a role in ameliorating colitis in adult mice. (**A**) Study design. Donors include mice with (2′-FL donor) or without (control donor) 2′-FL treatment. Feces were collected for three consecutive days from donor groups and pooled within groups for FMT to recipient mice after recipient mice received broad-spectrum antibiotic treatment and a subsequent 48-hour washout period. FMT was administered for three consecutive days, after which mice received no treatment for three consecutive days before treatment with DSS or water as a control. (**B**) Representative hematoxylin and eosin staining of colonic sections. (**C**) Colon injury and inflammation scores. (**D**) Quantification of TNF mRNA expression by RT-PCR. The average mRNA expression levels in the antibiotics + water group were set as 100%, and the mRNA expression level of each mouse was compared with the average. (**E**) Colonic sections were immunostained with an anti-ZO-1 antibody and a FITC-labeled secondary antibody (green). Nuclei were stained with DAPI (blue). Membrane (white arrows) and intracellular (yellow arrows) ZO-1 distributions are shown.

Because we found that 2′-FL pretreatment mitigated colitis in two mouse models of acute colitis, dextran sulfate sodium (DSS)-induced intestinal injury and inflammation, and 2,6,4-trinitrobenzenesulfonic acid (TNBS)-induced Th1-driven colitis in adult mice (Fig. S1), we leveraged the fecal microbiota transplantation (FMT) model to investigate the role of the 2′-FL-modulated gut microbial community on intestinal inflammation. FMT donor mice received 2′-FL in regular drinking water (2′-FL FMT) or regular water (control FMT). FMT recipient mice received antibiotic treatment to decrease the endogenous gut bacteria, followed by FMT and DSS-induced colonic injury and colitis (Fig. 2A). DSS treatment induced acute injury and colitis with massive colon ulceration, crypt damage, and severe inflammation with the injury/inflammation score of 17.18 ± 2.92, which was significantly mitigated by 2′-FL FMT (6.5 ± 1.01; *P* = 0.0021) but not control FMT (12.2 ± 2.44; *P* > 0.05) (Fig. 2B and C). DSS-induced mRNA expression of TNF, a proinflammatory cytokine, was significantly decreased by the 2′-FL FMT, as compared to the DSS and DSS plus control FMT groups (*P* = 0.0186 and *P* = 0.0126, respectively) (Fig. 2D). The distribution of a tight junctional protein, ZO-1, was detected by immunostaining. The 2′-FL FMT, but not the control FMT, prevented DSS-induced redistribution of ZO-1 from apical tight junctional complexes to the cytoplasmic compartment of colon epithelial cells in adult mice (Fig. 2E), indicating the protective roles of the 2′-FL-modified gut microbiota profile on the intestinal barrier. These results suggest that 2′-FL could lead to a balancing of the gut microbial community that exerts preventive effects on intestinal inflammation.

### Pantothenol is a novel 2′-FL secretory metabolite by *B. infantis*

**Fig 3:**
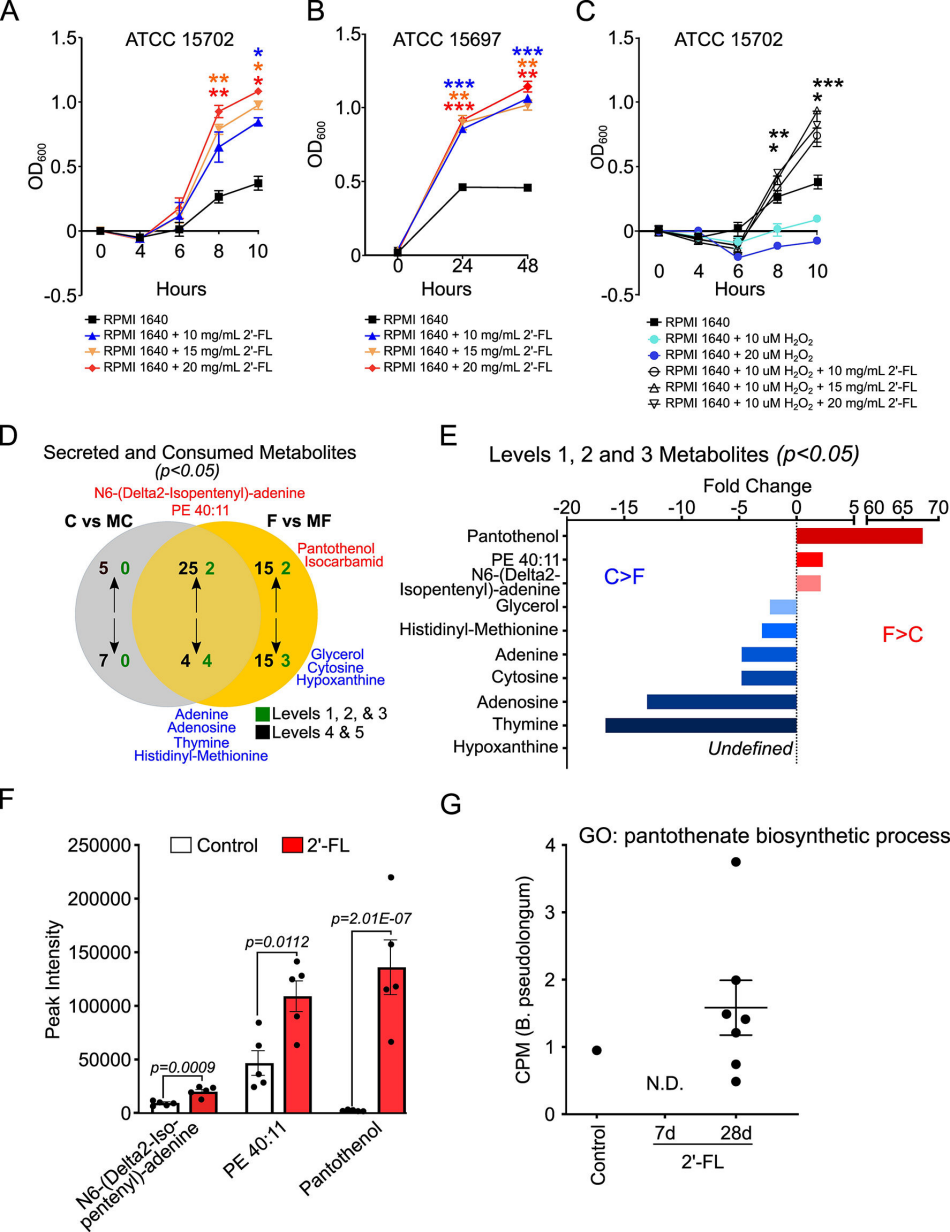
*Bifidobacterium infantis* growth and metabolism of 2′-fucosyllactose *in vitro*. (**A and B**) *B. infantis* strains ATCC 15702 (**A**) and ATCC 15697 (**B**) were grown in RPMI 1640 medium (no glucose) with and without supplementation with 2′-FL at indicated concentrations and OD_600_ was recorded. (**C**) Kinetics of ATCC 15702 growth in the presence of H_2_O_2_ with and without supplementation with 2′-FL at indicated concentrations. (**D**) *B. infantis* strain ATCC 15702 was cultured in reinforced clostridial medium with and without 2′-FL at 10 mg/mL for 7 hours (mid-logarithmic growth phase). Metabolites in the medium only (medium control, MC), medium with 2′-FL (MF), supernatant from *B. infantis* culture in MC (**C**), and supernatant from *B. infantis* in MF (**F**) were identified by metabolomics. Metabolite names in red are enriched (secreted), while those in blue are depleted (consumed). Levels 1, 2, and 3 metabolites (green) were identified with high confidence, while levels 4 and 5 were not and thus were not considered for further analysis. (**E**) Fold change for significant (*P* < 0.05) F vs C supernatant [secreted (red) and consumed (blue)] levels 1, 2, and 3 metabolites. (**F**) Normalized peak intensity values for the three secreted metabolites identified in panel **E** between 2′-FL and control supernatants, depicting actual abundance. (**G**) Abundance (CPM, counts per million) of the pantothenate biosynthetic process pathway for *Bifidobacterium pseudolongum* identified in mice feces. One data point represents one mouse (N.D., not detected). *P < 0.05, **P < 0.01, and ***P < 0.001 compared to control within the same time point. For panels A–C, asterisks are color coded to represent the condition that is significant, compared to the control (black). At least three independent bacterial culture experiments were performed.

To further investigate how 2′-FL exerts protective effects through regulation of the gut microbiota, we focused on *B. infantis* as a bacterial model. *B. infantis* possesses gene clusters for the metabolism of HMOs (24) and is present in healthy adults in low abundance (22, 23). Multiple *B. infantis* strains have been shown to be able to grow on 2′-FL as a sole carbon source (25). Consistent with this evidence, 2′-FL had significant effects on promoting the growth of *B. infantis* ATCC 15702 (Fig. 3A) and ATCC 15697 (Fig. 3B) in Roswell Park Memorial Institute (RPMI) medium (no glucose) compared to the controls. Interestingly, we found that 2′-FL was able to significantly improve the growth of *B. infantis* 15702 under oxidative stress when *B. infantis* 15702 was cultured in the presence of 2′-FL and H_2_O_2_ (*P* < 0.05) (Fig. 3C).

Next, we applied untargeted metabolomic analysis of bacterial culture supernatants to discover 2′-FL-derived metabolite secretions by *B. infantis* 15702 cultured in reinforced clostridial medium (RCM). By comparing metabolites in the control medium (MC), medium with 2′-FL (MF), control culture supernatant (C), and 2′-FL culture supernatant (F), we detected four secreted and seven consumed significant metabolites. These significant metabolites were annotated using the metabolite level identification system (i.e., validated, putative, and tentative annotations) (26) and are shown in Fig. 3D. Of the secreted metabolites, pantothenol and isocarbamid were unique to the 2′-FL consumption, and N6-(Delta2-Isopentenyl)-adenine and PE 40:11 were common. Of the consumed metabolites, four were common (adenine, adenosine, thymine, and histidyl-methionine) and three were unique to the 2′-FL medium (glycerol, hypoxanthine, and cytosine). There were no secreted or consumed metabolites unique to the control group. Of the three significant 2′-FL-derived secreted metabolites, pantothenol (alcohol analog of pantothenate; vitamin B5) was the most abundant (>65-fold change compared to control; Fig. 3E) and significantly higher in normalized abundance/peak intensity in the 2′-FL supernatants compared to the control supernatant (*P* = 2.01 × 10^−7^; Fig. 3F). Additionally, the pantothenate biosynthetic process for *Bifidobacterium pseudolongum* was enriched after 28 days of 2′-FL consumption (identified in all seven mice) compared to the control group (identified in one mouse) and the 2′-FL 7D group (no mice identified) (Fig. 3G). Our results identify the secretion of novel 2′-FL-stimulated *B. infantis* metabolites, especially pantothenol, thus contributing to our understanding of the mechanisms of HMO-regulated metabolic pathways in gut microbes.

### Pantothenate protects the intestinal epithelial barrier and prevents colitis in adult mice

Pantothenate from the diet and gut microbial metabolism is water soluble and can be absorbed by intestinal epithelial cells (27). Pantothenate is required for the biosynthesis of coenzyme A (CoA), which is involved in processes such as the citric acid cycle and lipid metabolism and has shown antioxidant effects due to the formation of glutathione peroxidase, which is essential to protect against free radical-induced apoptosis (28). Pantothenate has been found to accelerate dermal fibroblast migration and proliferation during wound healing (29). Notably, pantothenate levels are reduced in the gut of IBD patients (30). Therefore, we tested if pantothenate could preserve the mucosal barrier and prevent colitis in adult mice.

**Fig 4:**
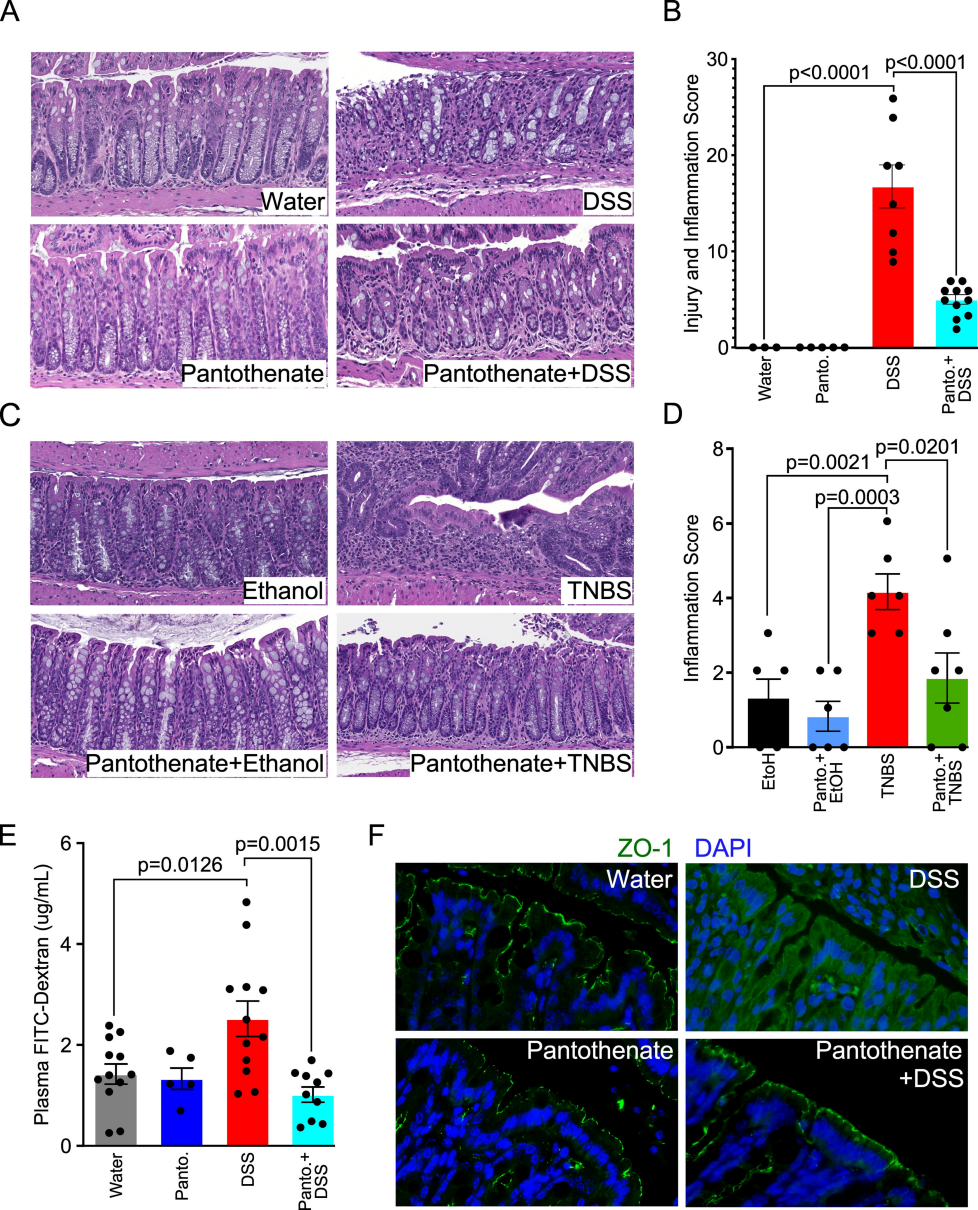
Pantothenate reduces colitis and preserves intestinal epithelial barrier in mice. Adult mice received either water (control) or pantothenate (Panto., 1 mM) in drinking water for 14 days prior to induction of colitis by DSS or TNBS. (**A and B**) Representative hematoxylin and eosin (H&E) staining of colon sections and colon injury and inflammation scores for mice with DSS treatment. (**C and D**) Representative H&E staining of colon sections and inflammation scores for mice with TNBS treatment. (**E**) Quantification of plasma FITC-Dextran in mice in the DSS experimental model. (**F**) Colonic sections from the DSS models were immunostained with an anti-ZO-1 antibody and a FITC-labeled secondary antibody (green).

Mice received pantothenate in drinking water for 2 weeks before induction of colitis. Compared to the intestinal injury and inflammation by DSS (score: 16.75 ± 2.23) and colitis by TNBS (score: 4.17 ± 0.48), pantothenate treatment significantly decreased DSS (score: 4.99 ± 0.49, *P* < 0.0001) (Fig. 4A and B) and TNBS-induced colitis (score: 1.86 ± 0.67, *P* = 0.0201) (Fig. 4C and D). To further characterize the effects of pantothenate on DSS-induced disruption of intestinal integrity, we performed an *in vivo* permeability assay to test intestinal barrier function. DSS-disrupted barrier function, as evidenced by increased FITC-dextran in the plasma was significantly decreased by pantothenate treatment (*P* = 0.0015, Fig. 4E). Furthermore, we determined the distribution of a protein marker of tight junction structure, ZO-1, using immunostaining. DSS-induced redistribution of this protein from the apical surface to the intracellular compartment was prevented by pantothenate co-treatment in (Fig. 4F).

**Fig 5:**
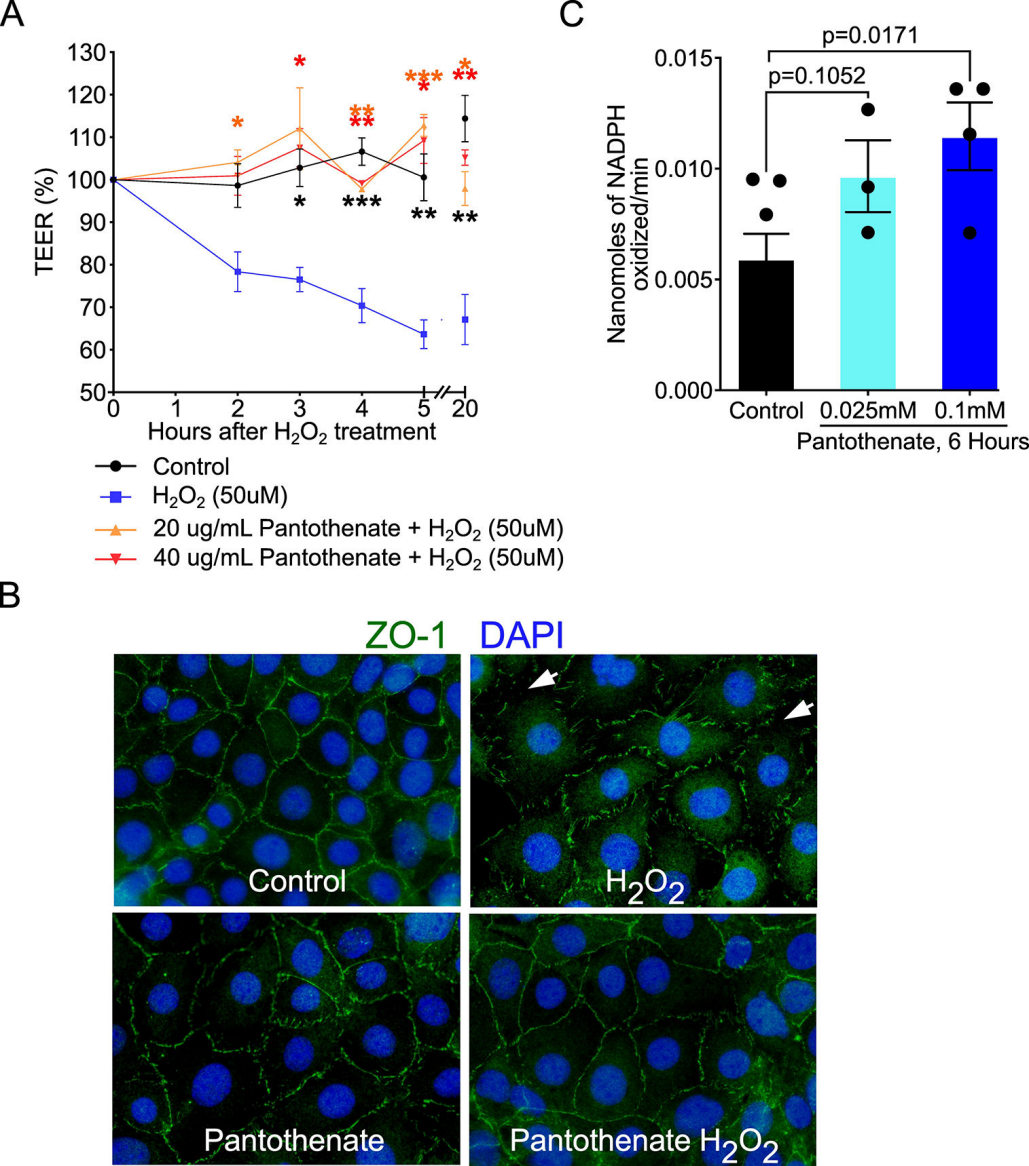
Pantothenate preserves the intestinal epithelial barrier and inhibits oxidative stress in intestinal epithelial cells. (**A**) Caco2 cells were pretreated with 20 or 40 µg/mL of D-calcium pantothenate for 2 hours before treatment with 50 µM of H_2_O_2_ for indicated times. Transepithelial electrical resistance was recorded. (**B**) YAMC cells were treated with H_2_O_2_ (20 µM) with and without co-treatment with pantothenate at 0.1 mM for 4 hours. Cells were fixed for immunostaining using an anti-ZO-1 antibody and a FITC-labeled secondary antibody (green). Nuclei were stained with DAPI (blue). White arrows represent the disruption of the tight junction. (**C**) YAMC cells were treated with pantothenate at 0.025 and 0.1 mM for 6 hours. Cellular lysates were collected for the GPX activity measured as nanomoles of NADPH oxidized/minute. *P < 0.05, **P < 0.01, and ***P < 0.001 compared to 50 µM H_2_O_2_ within the same indicated time point. For panel A, asterisks are color coded to represent the condition that is significant compared to H_2_O_2_ (blue). At least three independent bacterial culture experiments were performed.

We next studied how pantothenate could exert direct effects on intestinal epithelial cells to protect the intestinal barrier by using *in vitro* assays. We evaluated the effect of pantothenate on the oxidative stress-induced disruption of the epithelial barrier. H_2_O_2_ reduced the paracellular permeability in Caco2 cells measured by transepithelial electrical resistance (TEER) in a time-dependent manner, which was significantly reduced by pantothenate treatment (Fig. 5A). Furthermore, H_2_O_2_-induced redistribution of ZO-1 from apical tight junctional complexes to the cytoplasmic compartment of YAMC cells was prevented by pantothenate (Fig. 5B). We further studied how pantothenate reduces oxidative stress in YAMC cells by measuring the activity of glutathione peroxidase (GPX), an antioxidant enzyme involved in scavenging free radicals, resulting in a decrease in oxidative stress. Pantothenate at 0.1 mM significantly increased GPX activity in YAMC cells compared to control cells (*P* = 0.0171, Fig. 5C).

These results suggest that pantothenate plays a role in the preservation of epithelial integrity and prevention of colitis in adult mice, which may be through mitigating the oxidative stress associated with inflammation.

### 2′-FL-regulated microbial metabolic pathways are dysregulated in patients with UC

**Fig 6:**
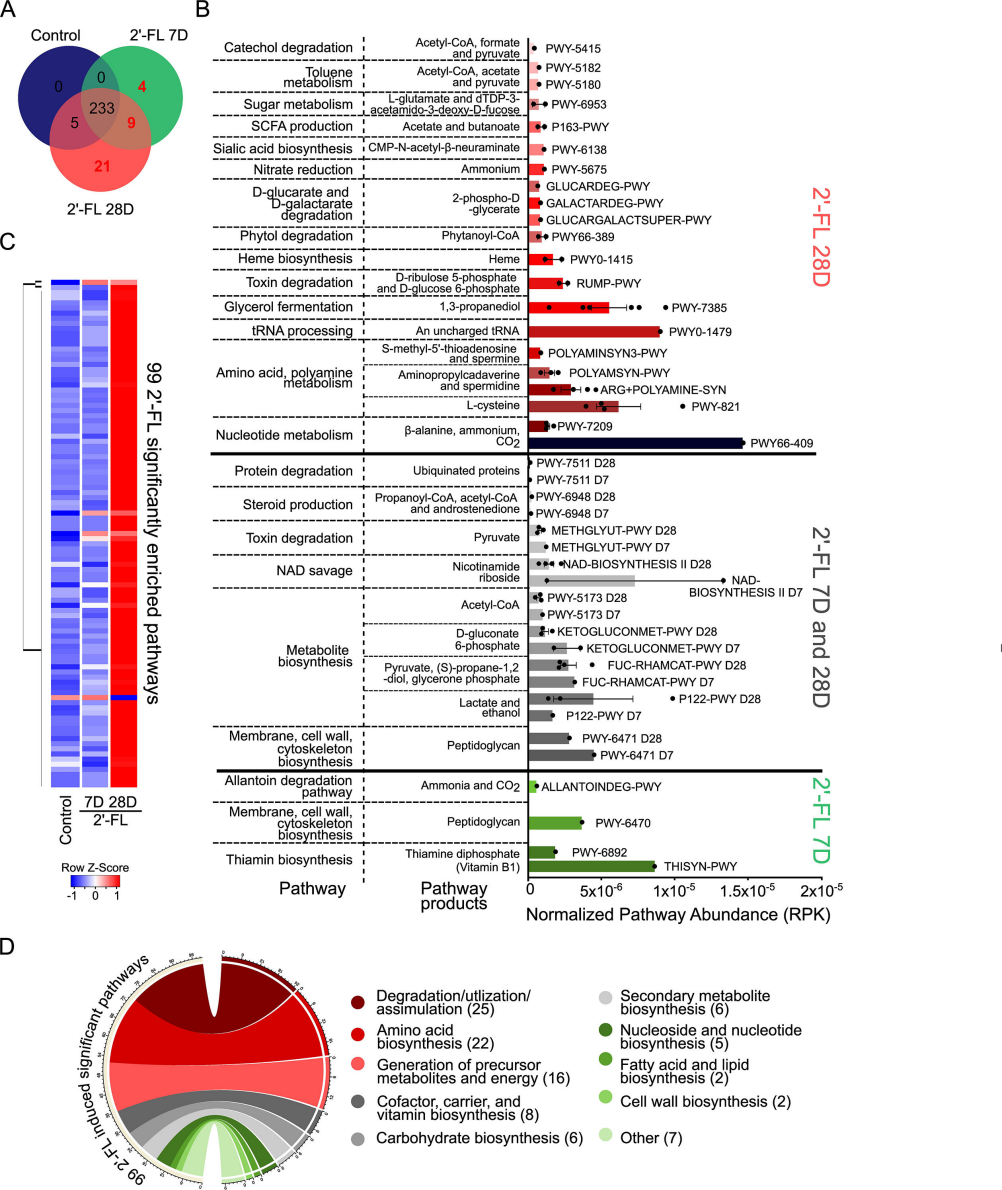
Unique and significantly enriched gut microbial metabolic pathways by 2′-FL consumption in adult mice. Adult mice received 2′-FL treatment as described in Fig. 1. (**A**) Microbial metabolic pathways identified by the HUMAnN3 program were compared between the control, 2′-FL 7D, and 2′-FL 28D groups. A total of 34 pathways were identified as uniquely detectable after 2′-FL consumption, but not in any control mice, including four pathways only after 7 days 2′-FL, 9 pathways after 7 or 28 days 2′-FL, and 21 pathways after 28 days 2′-FL. (**B**) Metabolic pathway abundance [bar blots, in reads per kilobase (RPK)] normalized by mapped reads per individual animal and the names of their respective pathway byproducts and products are illustrated for these 34 pathways. Pathways are grouped according to one of the three experimental group(s) they were identified in (from panel A) and are further organized into super pathway functions. (**C**) Of the 233 common pathways between all three study groups, 99 were significantly different in abundance (*P* < 0.05) in the 2′-FL 28D group (98 pathways) or the 2′-FL 7D and 28D groups (one pathway) compared to the control group. The 99 significantly enriched pathways are displayed in a hierarchically clustered heatmap to illustrate the changes in enrichment. (**D**) Functional categorization of the 99 significantly different pathways into the top 10 biological functions illustrated in a circus plot.

We further studied the impact of 2′-FL consumption on the gut microbial metabolic pathways in adult mice. Functional profiling was performed using the HMP Unified Metabolic Analysis Network (HUMAnN3) program, which profiles the presence/absence and abundance of microbial metabolic pathways (31). In total, 238, 246, and 268 total pathways were identified in the control, 2′-FL 7D, and 2′-FL 28D groups, respectively. By comparing these pathways presence among the three groups, in addition to 233 pathways that were common among all three groups, we identified 34 pathways only present after 2′-FL consumption: 4 pathways only in the 2′-FL 7D group, 9 pathways in the 2′-FL 7D and 28D groups, and 21 pathways only in the 2′-FL 28D group. Furthermore, there are five pathways that were identified in the control and 2′-FL 28D groups (Fig. 6A; Table S4). Further classification of the 34 pathways uniquely enriched by 2′-FL identified common super pathways and pathway products, with individual pathways (identified by BioCyc ID) abundance plotted for each group (Fig. 6B). Of the 34 2′-FL uniquely enriched microbial pathways, several produce metabolites and signaling molecules that have been found to be depleted in or to benefit IBD patients’ symptoms or ameliorate symptoms in rodent models of IBD, including thiamine (32), lactate (33), spermidine/spermine (34), acetate and butanoate (35), and fumarate (36).

Of the 233 common pathways, 99 were statistically different in abundance (*P* < 0.05) between the 2′-FL groups compared to the control group, the majority of which were enriched by 2′-FL consumption for 28 days as depicted by the heatmap (Fig. 6C). Of the 99 pathways, 98 pathways were significantly different between 28 days 2′-FL vs control, and one pathway was significantly different between 7 and 28 days 2′-FL vs control (Table S5). Classification of the 99 pathways by common functions identified that the majority were involved in degradation/utilization/assimilation (25) and amino acid biosynthesis (22), followed by generation of precursor metabolites and energy (16), cofactor, carrier, and vitamin biosynthesis (8), carbohydrate biosynthesis (6), secondary metabolite biosynthesis (6), nucleoside and nucleotide biosynthesis (5), fatty acid and lipid biosynthesis (2), cell wall biosynthesis (2), and others (7) (Fig. 6D) (Table S6). These results indicate that 2′-FL could shape the metabolic functions of gut microbes which in turn can benefit the host.

**Fig 7:**
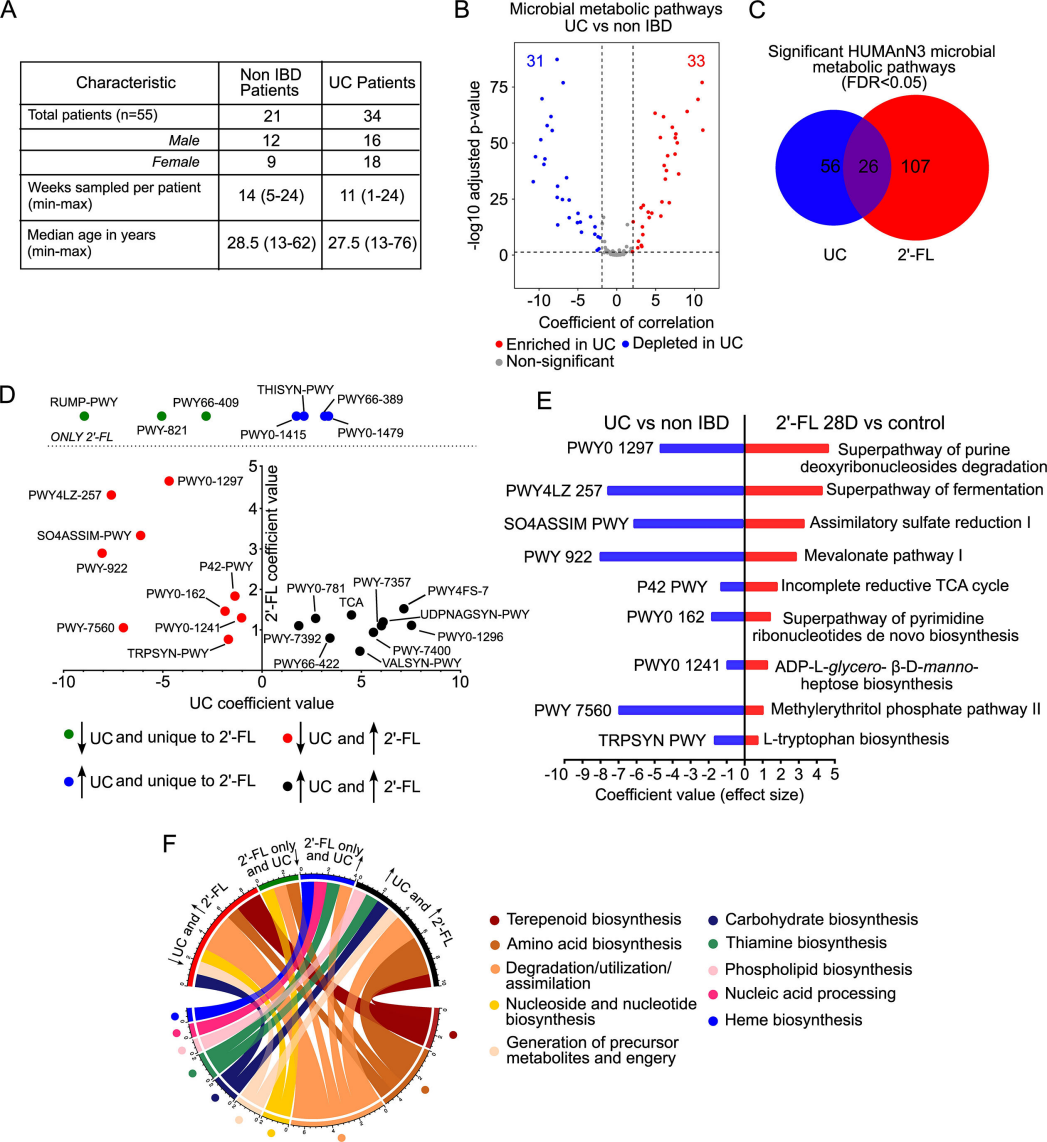
Microbial metabolic pathways that are altered in patients with UC are regulated by 2′-FL consumption in adult mice. (**A**) Table of characteristics for UC patients. Human stool samples were analyzed by WGS and the HUMAnN3 program. (**B**) Volcano plot comparing the significance of the coefficient of correlation (effect size) of the 372 microbial metabolic pathways between non-IBD and UC patients, including 33 pathways that were significantly positively correlated/enriched (red, correlation coefficient > 2 and FDR < 0.05) with ulcerative colitis, and 31 pathways that were significantly negatively correlated/depleted (blue, correlation coefficient < −2 and FDR < 0.05) with ulcerative colitis. (**C**) Comparison of significantly altered microbial metabolic pathways in UC patients (FDR < 0.05) and after 2′-FL consumption in adult mice (FDR < 0.05) identified 26 common differentially abundant pathways. (**D**) Plotting the coefficient of correlation for the 26 common ulcerative colitis and 2′FL-regulated pathways identified nine pathways depleted in UC but enriched by 2′FL (red), three pathways depleted by UC and only detected after 2′-FL consumption (green), four pathways enriched by UC and only detected after 2′-FL consumption (blue), and 10 pathways enriched by UC and 2′-FL (black). (**E**) Direct comparison of the coefficient of correlation (effect size) for the nine pathways enriched after 2′-FL 28D consumption in mice that are also depleted in UC patient fecal microbes. 2′-FL was compared to the control, and UC patients were compared to non-IBD patients. (**F**) Circos plot categorizing common super pathway functions for the four groups of pathways identified in panel D.

To reveal the relevance of 2′-FL-regulated microbial metabolic pathways to IBD, we utilized HUMANn3 to analyze whole-genome sequencing data of human fecal microbiota from 34 patients with UC and 21 non-IBD control subjects collected by the NIH Human Microbiome project, which is available in the online repository (https://ibdmdb.org/tunnel/public/HMP2/WGS/1818/products) (Fig. 7A). We identified 372 total microbial metabolic pathways in non-IBD and UC patients (Table S7), of which 82 pathways were significantly different between non-IBD and ulcerative colitis patients (FDR < 0.05) (Table S8). Further analysis of these 82 pathways showed that 33 pathways were significantly positively correlated/enriched with UC (correlation coefficient > 2 and FDR < 0.05), 31 pathways were significantly negatively correlated/enriched with UC (correlation coefficient < −2 and FDR < 0.05) (Fig. 7B). Comparison of the 99 statistically different in abundance pathways by 2′-FL and 34 unique pathways to 2′-FL in mice with the 82 statistically different pathways in human samples identified 26 pathways that were both altered by 2′-FL consumption and in UC (Fig. 7C) (Table S9). Plotting the 26 common pathways based on the coefficient of correlation for 2′-FL (*y*-axis) vs UC (*x*-axis) divided the pathways into four distinct cohorts: unique to 2′-FL and depleted in UC (three pathways, green), unique to 2′-FL and enriched in UC (four pathways, blue), enriched in 2′-FL and UC (10, black), and enriched in 2′-FL and depleted in UC (nine pathways, red) (Fig. 7D) (Table S10). A direct comparison of the nine 2′-FL-enriched and UC-depleted pathways identified microbial functions that 2′-FL can restore in UC patients (Fig. 7E), while a biological function-based categorization of the 26 shared pathways identified general functions that pathways are involved in (Fig. 7F). 2′-FL-enriched, or unique, pathways and UC-depleted pathways are primarily involved in the generation of precursor metabolites and energy (1), degradation/utilization/assimilation (4), and amino acid biosynthesis (2), nucleoside and nucleotide biosynthesis (2), carbohydrate biosynthesis (1), and terpenoid biosynthesis (2). In contrast, 2′-FL-enriched, or unique, pathways and UC-enriched pathways are primarily involved in amino acid biosynthesis (3), degradation/utilization/assimilation (3), thiamine biosynthesis (2), generation of precursor metabolites and energy (1), terpenoid biosynthesis (1), phospholipid biosynthesis (1), nucleic acid processing (1), and heme biosynthesis (1). These results demonstrate the potential application of 2′-FL for IBD prevention and treatment.

## DISCUSSION

HMOs have shown multiple beneficial effects in infancy and beyond. However, the biological consequences of the HMO-modified gut microbial profile on intestinal homeostasis, especially under inflammatory conditions, and the corresponding mechanisms of the action remain largely unknown. To address this knowledge gap, this work demonstrated that the 2-FL-modulated gut microbial community plays significant roles in mitigating intestinal injury and colitis in adult mice and identified a metabolic mechanism involved in this effect. By using *B. infantis* as a 2′-FL consumption bacterial model, we found pantothenol as a novel 2′-FL-derived secreted metabolite that can maintain intestinal barrier integrity and protect against colon injury and inflammation.

To further elucidate how 2′-FL regulates *Bifidobacteria* function, we employed untargeted metabolomics to discover three novel 2′-FL-derived secreted metabolites by *B. infantis* that, to our knowledge, have not been previously reported. Of these, pantothenol was the most abundant and was thus further pursued for functional studies. To our knowledge, research on pantothenol function in the gastrointestinal tract appears to be limited. In agreement with studies on human dermal fibroblasts (29), we found pantothenate could reduce oxidative responses, protect the intestinal barrier, and prevent colitis. Furthermore, a pantothenate biosynthesis pathway in *B. pseudolongum* species was enriched by 2′-FL consumption in mice (Fig. 3G). Interestingly, we found three microbial pathways related to pantothenate production, which were numerically lower in UC patients compared to non-IBD controls (Table S11). Thus, pantothenate production may serve as a mechanism of 2′-FL-directed microbial metabolism for the prevention of intestinal inflammation.

In addition to pantothenol, we found several previously unrecognized 2′-FL metabolites. Phosphatidylethanolamine (PE) PE:40 is a lipid that is predominantly found in both human and bovine milk, enriched in the milk fat globule membrane and a source of arachidonic acid and docosahexaenoic acid (37). N6-(Delta2-Isopentenyl)-adenine, a modification of adenine, was the third upregulated 2′-FL-derived secreted metabolite. While this metabolite is found in plant tissue as a component of tRNA, to our knowledge, its function in the gastrointestinal tract is unclear. Therefore, we do not exclude the potential effects of additional 2′-FL metabolites that contribute to the prevention of colitis through 2′-FL-modulated gut microbial metabolism. It should be noted that in the murine HUMAnN3 data, fumarate was found to be produced by five pathways in the enriched *B. pseudolongum* species (Fig. S2). While it is not known from our data if it was secreted, fumarate has been found to ameliorate inflammatory responses in rodent colitis models and maybe another microbe-mediated mechanism by which 2′-FL protects against colitis.

While *B. infantis* has been identified in adults (22, 23), *Bifidobacteria* abundance is reduced in adults with UC (38) and CD (39) compared to non-IBD counterparts, particularly *Bifidobacterium longum* (40), suggesting a decline in *Bifidobacteria*-mediated dysbiosis in IBD. In this study, we found that 2′-FL enriched for *Bifidobacterium*. Furthermore, these data show that 2′-FL was able to significantly improve the growth of *B. infantis* under oxidative stress. As oxidative stress is a hallmark of IBD (41), this suggests that 2′-FL may enable *B. infantis* growth despite the heightened oxidative stress environment.

We further revealed that some dysregulated microbial metabolic pathways that are significantly different between non-IBD individuals and patients with UC are significantly regulated by 2′-FL in mice, indicating the potential benefits of metabolism of 2′-FL on IBD. It should be noted that alteration of microbial metabolic pathways in IBD should be associated with dysbiosis and the functional changes of the gut microbiota under inflammatory conditions. The impact of 2′-FL on metabolism in IBD might be related to the regulation of the gut microbial balance, especially the growth of HMO-utilizing bacteria, and the direct effects on promoting metabolic pathways in bacteria involved in 2-FL-mediated metabolite production. Of the 2′-FL-regulated microbial metabolic pathways that are dysregulated in UC, several appear to be interesting targets with potential clinical relevance. For example, the tryptophan biosynthesis pathway (TRPSYN-PWY) was depleted in UC patients but enriched by 2′-FL. Tryptophan is an essential amino acid that is the sole precursor for serotonin production by gut microbes (42) and can be catabolized into a multiplicity of catabolites that regulate mucosal homeostasis and gut immune cells (43). Moreover, microbial pathways involved in thiamine biosynthesis (PWY-7357 and THISYN-PWY) are essential for energy metabolism, as thiamine is a cofactor for a variety of enzymes involved in carbohydrate metabolism and reduction of oxidative stress. The enrichment of thiamine biosynthesis pathway abundance by 2′-FL and in UC patients may be due to the need to produce thiamine to compensate for disrupted host energy homeostasis in a dysbiotic disease state. Interestingly, two studies found an improvement in fatigue when patients were given high-dose oral thiamine compared to placebo (32). Identification of these coupled pathways uncovers the potential of 2′-FL for mitigating the altered microbial metabolism in IBD patients.

Collectively, we identified a biological mechanism by which 2′-FL-regulated gut microbial community protects the intestinal barrier and decreases the susceptibility to intestinal inflammation through the production of beneficial metabolites. We also provided novel evidence to identify microbial metabolic pathways regulated by 2′-FL consumption that may compensate for the disturbance of microbial metabolism in IBD. Thus, this research provides new insight into developing 2′-FL as a safe and tolerable strategy for protecting the intestinal integrity and inhibiting colitis for patients with IBD.

## MATERIALS AND METHODS

### Mouse treatment

Male and female wild-type mice on C57BL/6 and Balb/c background were group housed and maintained with a 12-hour light-dark cycle. Food (PicoLab Laboratory Rodent Diet L50D diet, LabDiet) and water were provided *ad libitum* for all experiments.

Mice at the age of 6–8 weeks received 2′-FL [synthesized as previously described (44)] at 1 mg/mL in the drinking water for 7, 28, or 35 days before induction of colitis or serving as donors for the FMT experiments. Mice at the age of 6–8 weeks received D-calcium pantothenate at 1 mM (Cat#243300050, Fisher Scientific) in drinking water for 14 days. Mice given drinking water were used as a control. Baseline mouse daily water intake and body weight before and after treatments were monitored throughout the experiments.

### FMT

Recipient mice received antibiotic treatment by oral administration of 200 mg/kg of body weight/mouse (for each antibiotic) of cefoperazone (Cat#C-660-5, Gold Biotechnology, Inc.), metronidazole (Cat#M81000, Research Products International), ampicillin sodium (Cat#A-301-5, Gold Biotechnology, Inc.), and neomycin sulfate (Cat#N-620-10, Gold Biotechnology, Inc.) once a day for four consecutive days, during which time mice were housed in autoclaved cages with autoclaved food and sterile water, which were changed daily. Mice receiving antibiotics were allowed a washout period of 48 hours (no treatment) in sterile cages before FMT (45).

Mice receiving 2′-FL for 35 days served as the 2′-FL-treated donors (2′-FL-donor) and mice receiving water as the control donors (control-donor). Fecal slurry samples were prepared from pooled stool from 2′-FL-donor (*N* = 3–5) and control-donor (*N* = 3–5) mice. Feces from individual mice (250 mg feces/mouse) were resuspended and vortexed in sterile PBS with 0.05% cysteine-HCl and pelleted by sequential centrifugation (500 *g* for 5 minutes, 800 *g* for 3 minutes, and 2,000 *g* for 5 minutes). The supernatant was filtered through a 70 µm filter to remove particulates and concentrated by centrifugation at 5,000 *g* for 20 min. The pellets were then resuspended in PBS with 0.05% cysteine-HCl (pellets from 250 mg feces to 1 mL PBS) for immediate oral gavage into recipient mice. Recipient mice received 100 µL of fecal slurry supernatant samples from 2′-FL or control donors by oral gavage once a day for three consecutive days. Subsequently, recipient mice were allowed 72 hours to promote bacteria colonization before colitis was induced. Antibiotic-treated mice without FMT were used as a control.

### Colitis induction and assessment

For the induction of colitis, mice on the C57BL/6 background were treated with 3% dextran sulfate sodium ( Cat#160110, molecular weight of 36–50 kDa, MP Biomedical, Solon, OH, USA) in drinking water. Mice were euthanized on day 4 of DSS treatment. For mice receiving 2′-FL or pantothenate treatment, 2′-FL and D-calcium pantothenate were provided by oral gavage during DSS treatment to keep 2′-FL and pantothenate intake consistent with pre-treatment consumption.

Colitis induced by 2,6,4-trinitrobenzenesulfonic acid (Cat#92822, Millipore Sigma) in mice on the Balb/c background was performed by intrarectal injection of 100 mL of 70 mM TNBS in 50% ethanol. Control mice received 100 mL of 50% ethanol intrarectally. Mice were euthanized 4 days after ethanol or TNBS treatment.

Paraffin-embedded tissue sections of Swiss-rolled whole colon were stained with hematoxylin and eosin for light-microscopic examination to assess colon injury and inflammation. Samples from the entire colon were examined by a pathologist blinded to treatment conditions. DSS-induced colitis was evaluated by a modified combined scoring system (46) including A. inflammation (scale of 0–3), B. depth of inflammation (scale of 0–3), C. percentage of area involved by inflammation (0–4), D. depth of crypt damage (scale of 0–3), and E. percentage of area involved by crypt damage (0–4). The total score = (A + B) × C + D × E. The scoring system used to assess TNBS-induced colitis included A. lamina propria mononuclear cell and polymorphonuclear cell infiltration, B. enterocyte loss, C. crypt inflammation, and D. epithelial hyperplasia, which were scored from 0 to 3. The total score = A + B + C + D.

### Microbial metabolic profiling using the HMP Unified Metabolic Analysis Network program

Mouse fecal metagenomics data were analyzed through the HUMAnN3 program to profile microbial metabolic pathways. HUMAnN3 pathway data were normalized by reads per kilobase and mapped reads per individual animal. Common and unique pathways between groups were identified using InteractiVenn, (47) and the 233 common pathways were further analyzed using the MaSaLin package (48) in R studio to identify the significantly different pathways (FDR < 0.05). Microbial metabolic pathways (identified by pathway names/BioCyc ID) were used to search the MetaCyc database (49) and to identify super pathways and pathway metabolite products and byproducts. Heatmap of significant pathways was generated using gplots package heatmap.2 function, hierarchical clustering average method. Circos plots were generated using the circlize library in R studio.

We used HUMAnN3 program microbial pathway abundance data for the NIH Human Microbiome WGS sequencing data set and patient metadata from the online repository (https://ibdmdb.org/tunnel/public/HMP2/WGS/1818/products). In this data set, 1,638 total stool samples (more than one sample was collected from each individual) with metagenomics analysis were collected, including 40 patients with ulcerative colitis and 27 non-IBD individuals. Patients who were excluded from this study were less than 12 years old, receiving antibiotics in the past 6 months or since the last stool sample collection for the study, being treated with an anti-TNF therapy, chemotherapy, or had immunosuppressants including oral corticosteroids, cancer, immune-mediated diseases, including rheumatoid arthritis, lupus, type 1 diabetes mellitus, acute gastrointestinal infection, or irritable bowel syndrome. This resulted in including 34 ulcerative colitis patients and 21 non-IBD individuals.

Human microbial pathway data (deposited data were normalized by the cpm method) were analyzed using the MaSaLin2 package (48) in R studio (https://huttenhower.sph.harvard.edu/maaslin/). For analysis, the non-IBD group was set as the reference, diagnosis as a fixed effect, and patient ID as a random effect to account for variability over the weeks sampled per patient (1–24 weeks of stool sampling per patient). Functional annotations using GO terms were performed through the One Codex database.

### *In vivo* intestinal epithelial cell permeability assay

FITC-conjugated dextran dissolved in water (4,000 mol wt., Cat#FD4-100MG, Sigma) was administered rectally to mice at 2 mg/10 g body weight. Whole blood was collected via the tail vein 2 hours after FITC-dextran administration. Fluorescence intensity in plasma was analyzed using a plate reader. The concentration of FITC-dextran in plasma was determined by comparing it to the FITC-dextran standard curve.

### Bacterial culture

*B. infantis* strains 15702 and 15697 were purchased from ATCC. Bacteria from frozen stocks were cultured in reinforced clostridial medium (Cat#DF1801-17-3, ATCC) with 180 RPM at 37°C until OD_600_≈ 1 for experiments. *B. infantis* 15702 was cultured in sterile glass tubes with rubber stoppers that were heat sealed with a flame, and *B. infantis* 15697 was cultured in sterile Eppendorf tubes that were paraffin sealed and cultured in a 2.5 L anaerobic jar with the AnaeroPack System (Cat#23-246-376).

For *in vitro* growth experiments, *B. infantis* 15702 and 15697 were inoculated in RPMI 1640 medium (no glucose) supplemented with 10, 15, or 20 mg/mL of 2′-FL (1.0%, 1.5%, or 2.0%, wt/vol, respectively). To study the effects of 2′-FL on oxidative stress, ATCC 15702 was cultured in 10 or 20 µM of H_2_O_2_. ATCC 15697 did not numerically nor statistically respond to H_2_O_2_ (data not included in this manuscript). OD_600_ was recorded. Independent culture experiments were performed at least three times.

### Cell culture

A mouse colonic epithelial cell line, young adult mouse colonic (YAMC) epithelial cells, was isolated from the mouse harboring a thermolabile simian virus 40 (SV40) large T antigen from an SV40 strain, tsA58.21 under the control of an interferon (IFN)-γ-inducible H-2Kb promoter at the permissive temperature (33°C) (50). YAMC cells were maintained in Roswell Park Memorial Institute 1640 medium supplemented with 5% fetal bovine serum (FBS), 5 U/mL murine IFN-γ, 5 µg/mL insulin, 5 µg/mL transferrin, 5 ng/mL selenous acid, and 100 U/mL penicillin and streptomycin at 33°C with 5% CO_2_.

The human colonic adenocarcinoma cell lines, Caco-2 (HTB-37, American Type Culture Collection, Rockville, MD, USA), were maintained in Dulbecco’s modified Eagle’s medium (DMEM) supplemented with 10% FBS and 100 U/mL penicillin and streptomycin at 37°C with 5% CO_2_.

### GPX activity assay

YAMC cells under the culture conditions were treated with D-calcium pantothenate (0.025 or 0.1 mM) for 6 hours before collection. Cells were rinsed with PBS, pelleted, and resuspended in RIPA buffer containing protease and phosphatase inhibitors, sonicated followed by pelleting of cells and collection of the supernatant. Briefly, the GPX activity assay was performed from cell lysates using a coupled assay with hydrogen peroxide as previously reported (38). The reaction mixture consisted of 50 mM potassium phosphate buffer (pH 7), 1 mM EDTA, 1 mM NaN_3_, 0.2 mM NADPH, 1 E.U./mL GSSG-reductase, 1 mM GSH, and 0.25 mM H_2_O_2_ in a total volume of 1 mL. All reagents except the enzyme source and peroxide were combined at the beginning of each day. Protein lysates were added to 0.8 mL of the above mixture and incubated at room temperature for 5 minutes before the initiation of the reaction with the addition of 0.1 mL peroxide solution. Absorbance at 340 nm was recorded for 5 minutes, and GPX activity was calculated from the slope as micromolar NADPH oxidized per minute. Experiments were independently performed three times.

### TEER measurement

Caco2 cells were seeded in transwells (Cat#3413, Corning Incorporated) and grown to TEER as 200–400 ohms, using EVOM^2^ Epithelial Voltohmmeter (World Precision Instruments, Inc.). After serum starvation in DMEM containing 0.5% FBS and 1% penicillin/streptomycin overnight, cells were pretreated with D-calcium pantothenate at 20 and 40 µg/mL for 2 hours followed by H_2_O_2_ treatment (50 µM) for 20 hours. Baseline TEER reading (0 hours) was performed before treatment, and subsequent readings were performed after the addition of H_2_O_2_. Readings were taken in three locations for each well and averaged for each time point. Experiments were independently performed three times.

### Data analysis and statistics

All data are presented as mean ± standard error of the mean. A *P* < 0.05 was defined as statistically significant. Statistical significance was determined using one-way ANOVA analysis followed by Tukey’s multiple comparisons test or unpaired *t* test for comparing data from two samples using GraphPad Prism 9.0 (GraphPad Software, Inc. San Diego, CA, USA). For microbial sequencing data analysis, significance for comparison between groups was set at *P* < 0.05. For the PCoA, a PERMANOVA analysis was performed. Analysis of the HUMAnN3 data for the mouse and human data sets was performed using the MaSaLin2 package, with significance set at FDR < 0.05. Metabolomics data significance was set at FDR < 0.05.

Group assignment and mouse number in each group for colitis models and methods for mouse fecal microbiota analysis, untargeted metabolomic analysis, immunostaining, and RT-PCR assay are provided in Supplementary Methods.

## Data Availability

The untargeted metabolomics data are available at the NIH Common Fund’s National Metabolomics Data Repository (NMDR) website, the Metabolomics Workbench, https://www.metabolomicsworkbench.org, where it has been assigned Study ID ST002737. The data can be accessed directly via its Project DOI: http://dx.doi.org/10.21228/M8Q42J. The murine fecal microbiome sequencing data have been deposited in the National Center for Biotechnology Information Sequence Read Archive with the BioProject accession code PRJNA986525. The human UC and non-IBD HUMAnN NIH data set is available in The Inflammatory Bowel Disease Multi’omics Database: https://ibdmdb.org/tunnel/public/HMP2/WGS/1818/products. All remaining data are available in the main text or supplementary materials.

## SUPPLEMENTAL MATERIAL

Supplemental figures mbio.00298-24-s0001.pdf Figures S1 and S2.

Supplemental text mbio.00298-24-s0002.docx Supplemental methods.

Supplemental tables mbio.00298-24-s0003.xlsx Tables S1-S11.

ASM does not own the copyrights to Supplemental Material that may be linked to, or accessed through, an article. The authors have granted ASM a non-exclusive, world-wide license to publish the Supplemental Material files. Please contact the corresponding author directly for reuse.
