# Supplementary material for: A human milk oligosaccharide prevents intestinal inflammation in adulthood via modulating gut microbial metabolism
Source: mBio. 2024 Mar 5;15(4):e00298-24. doi: 10.1128/mbio.00298-24 (PMC11005405; doi:10.1128/mbio.00298-24)
Supplement: Supplemental figures — Figures S1 and S2. [file mbio.00298-24-s0001.pdf]

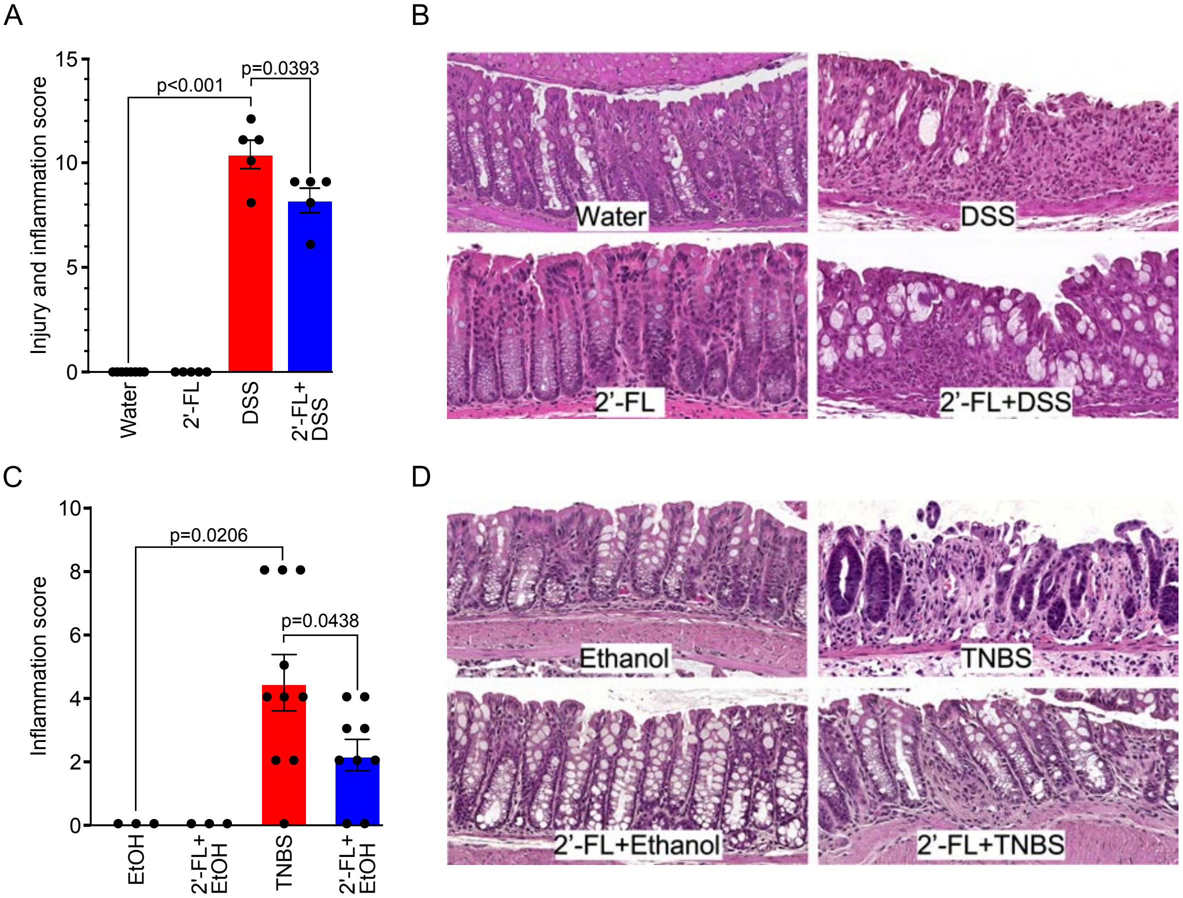

Supplemental Figure 1. 2'-FL ameliorates intestinal injury and inflammation in DSS and TNBS treated adult mice. (**A-B**) Mice pre-treated with 2'-FL (1 mg/mL) for 4 weeks prior to 3% DSS administration via drinking water for 4 days, then mice were euthanized. Colonic sections were prepared for H &E staining. Injury and inflammation scores (A) and representative H&E images (B) are shown. (**C-D**) Mice pre-treated with 2'-FL (1 mg/mL) for 4 weeks prior to rectal injection of TNBS or ethanol (EtOH) as a control. Mice were euthanized 4 days after TNBS treatment and colonic sections were prepared for H &E staining. Inflammation scores (C) and representative H&E images (D) are shown.

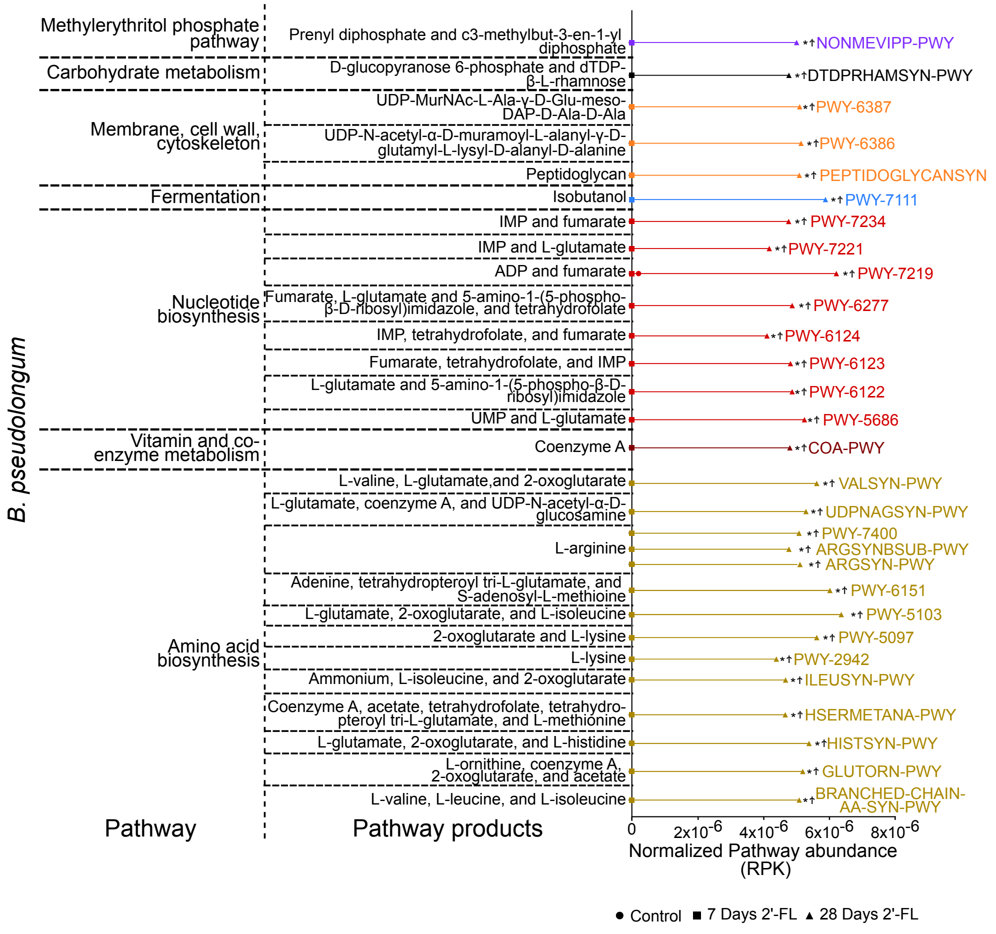

Supplemental Figure 2. Functional analysis of the metabolic pathways in *Bifidobacterium pseudolongum* from 2'-FL-treated mice using the HUMAnN3 program. Mice were treated with 2'-FL as described in Fig 1. The mean abundance (in normalized reads per kilobase (RPK)) of statistically upregulated microbial metabolic pathways (P<0.05 for at least one comparison) in *B. pseudolongum* in the 2'-FL 28 days group (triangle) compared to the control (circle) and the 2'-FL 7 days groups (square). MetaCyc pathway ID's (right of line graphs) were grouped into similar functional categories including amino acid biosynthesis, vitamin and co-enzyme metabolism, nucleotide biosynthesis, fermentation, membrane, cell wall and cytoskeleton, carbohydrate metabolism and methylerythritol phosphate pathway. *Indicates P < 0.05 for 28 days 2'-FL vs control, while † indicates P < 0.05 for 28 days 2'-FL vs 7 days 2'-FL. There were no statistically different pathways for 7 days 2'-FL vs control group. The abundance for each pathway is related to the mean abundance of gene copies for compositor enzymes within a sample.
