# Supplementary material for: A human milk oligosaccharide prevents intestinal inflammation in adulthood via modulating gut microbial metabolism
Source: mBio. 2024 Mar 5;15(4):e00298-24. doi: 10.1128/mbio.00298-24 (PMC11005405; doi:10.1128/mbio.00298-24)
Supplement: Supplemental text — Supplemental methods. [file mbio.00298-24-s0002.docx]

**SUPPLEMENTARY METHODS**

**Group assignment for colitis models**

Mice were randomly assigned to the following groups. (1) The effects of 2’-FL on DSS-induced intestinal injury and inflammation in C57BL/6 mice: Water group (n=8), DSS group (n=5), 2’-FL-28 day group (n=5), and 2’-FL-28 day + DSS group (n=5). (2) The effects of 2’-FL on TNBS-induced colitis in Balb/c mice: Ethanol group (n=3), TNBS group (n=10), 2’-FL-28 day + Ethanol group (n=3) and 2’-FL-28 day + TNBS group (n=9). (3) The effects of 2’-FL-regulated gut microbial community on DSS-induced intestinal injury and inflammation in C57BL/6 mice: Antibiotics + water group (n=10), antibiotics + DSS group (n=11), control FMT group + water (n=3), 2’-FL FMT + water group (n=3), control FMT + DSS group (n=6), and 2’-FL FMT + DSS group (n=8). The effects of pantothenate on DSS-induced intestinal injury and inflammation in C57BL/6 mice: Water group (n=3), DSS group (n=8), pantothenate group (n=5), pantothenate + DSS group (n=11). (5) The effects of pantothenate on TNBS-induced colitis in Balb/c mice: Ethanol group (n=6), TNBS group (n=6), pantothenate + ethanol group (n=6). pantothenate + TNBS group (n=7).

**Mouse fecal microbiota analysis**

C57BL/6 mice were randomly separated into three groups that received regular drinking water (n=5) or 2’-FL at 1 mg/ml in the drinking water for 7 days (n=5) or 28 days (n=7). Fecal samples were saved in DNA stabilization buffer for whole genome shotgun (WGS) sequencing by Transnetyx, Inc. (Cordova, TN). Briefly, DNA extraction was performed using the Qiagen DNeasy 96 PowerSoil Pro QIAcube HT extraction kit and protocol, and resulted in inhibitor-free, high molecular weight genomic DNA. Library preparation was performed using the KAPA HyperPlus library preparation protocol. After quality control, the Illumina NovaSeq instrument and protocol were used for sequencing. Libraries were sequenced using the shotgun sequencing method (for a depth of 2 million 2x150 bp read pairs) to enable species and strain level taxonomic resolution. Unique dual indexed (UDI) adapters were used to ensure that reads and/or organisms are not mis-assigned. Sequencing data was uploaded on to the One Codex analysis software^24^ and analyzed against the One Codex database which consists of >115K whole microbial reference genomes, including 62K distinct bacterial genomes, using K-mer based classification. The mouse genome was used to screen out host reads. Sequencing artifacts were filtered out, and species-level relative abundance was estimated based on sequencing coverage and depth across every available reference genome.

**Untargeted metabolomic analysis**

*B. infantis* 15702 were inoculated in RCM at 37°C with or without supplementation with 10 mg/mL of 2’-FL (1.0% w/v) for 7 hours. Bacteria were pelleted and supernatants were filtered (0.2 micron) and stored at -80ºC prior to sample preparation. Cultured RCM medium and 10 mg/mL 2’-FL supplemented RCM medium without bacteria inoculation served as controls. Experiments were repeated 2-3 times for collecting five biological replicates from each group. Culture supernatants were analyzed via liquid chromatography-high resolution mass spectrometry (LC-HRMS and LC-HRMS/MS)-based metabolomics using previously described methods^29,30^. Optima grade LC-MS solvents and chemicals for the mass spectrometry analyses were obtained from Thermo Fisher Scientific (Waltham, MA). Briefly, proteins were precipitated, and supernatants used for MS characterization. Insoluble material was removed from samples. The retention times and peak areas of the isotopically labeled standards were used to assess data quality. Global untargeted analyses were performed using previously published reverse phase liquid chromatography (RPLC) methods^31,32^. Mass spectrometry analyses were performed in positive ion mode with the parameters as previously published^31,32,33^ except for several modifications.

For data processing and analysis, the acquired RPLC-HRMS raw data from five biological replicates from each group were imported, processed, normalized, and reviewed using Progenesis QI v.3.0 (Non-linear Dynamics, Newcastle, UK). Data were normalized to all features and cleaned by removing spectral features >25% CV in the pooled QC samples. In these studies, no samples were identified as outliers. Statistical analyses were performed in Progensis QI using variance stabilized measurements achieved through log normalization to calculate p-values by one-way analysis of variance (ANOVA) test. Significantly changed metabolites were chosen with the criteria p≤0.05 and |FC| > 2. Tentative and putative annotations were determined by using accurate mass measurements (<5 ppm error), isotope distribution similarity, and fragmentation spectrum matching database searches against Human Metabolome Database (HMDB)^34^, METLIN^35^, and the Center for Innovative Technology’s in-house library. Annotations (Level 1-3)^36^ were determined for all significant changed metabolites with a match to any of our searched libraries or databases. The metabolite annotations from RPLC-HRMS/MS positive analysis are represented in Supplementary Table 11.

**Immunostaining**

Paraffin-embedded colon tissue sections were deparaffinized followed by antigen retrieval by placement in a pressure cooker (pH 6.2) for 20 minutes. Sections were blocked using 10% goat serum for 1 hour at room temperature. Cultured cells were fixed with 4% paraformaldehyde (Cat #15710, Electron Microscopy Sciences) overnight at 4°C and blocked using 5% goat serum in PBS containing 3% Triton X-100 for 1 hour at room temperature. Slides were incubated with a rabbit anti-mouse ZO-1 antibody (61-7300, Invitrogen Life Technologies) overnight at 4°C, followed by a FITC-labeled goat anti-rabbit IgG antibody (4412S; Cell Signaling Technology) at room temperature for 2 hours. DAPI staining was performed for 5 minutes. Sections were then mounted using mounting medium (Cat#0100-01, SouthernBiotech). Slides were observed using Lecia DM IRB inverted microscope and images were recorded using an Echo/Revolve (CD230RevA) camera or scanned using the Apiro Versa 200 platform.

**RT-PCR assay**

Total RNA was extracted from mouse colon tissue using the Qiagen RNeasy mini kit (Cat#74104; Qiagen). RNA samples were reverse transcribed into cDNA using the High-Capacity cDNA Reverse Transcription kit (Cat#4368814, Thermo Fisher Scientific). RT-PCR performed using the Taqman method using primers for mouse TNFα (Mm00443259, Applied Biosystems) and GAPDH (4352339, Applied Biosystems). The relative abundance of GAPDH mRNA was used to normalize levels of the mRNAs of interest. All cDNA samples were analyzed in duplicate.
